# Mutation of Growth Arrest Specific 8 Reveals a Role in Motile Cilia Function and Human Disease

**DOI:** 10.1371/journal.pgen.1006220

**Published:** 2016-07-29

**Authors:** Wesley R. Lewis, Erik B. Malarkey, Douglas Tritschler, Raqual Bower, Raymond C. Pasek, Jonathan D. Porath, Susan E. Birket, Sophie Saunier, Corinne Antignac, Michael R. Knowles, Margaret W. Leigh, Maimoona A. Zariwala, Anil K. Challa, Robert A. Kesterson, Steven M. Rowe, Iain A. Drummond, John M. Parant, Friedhelm Hildebrandt, Mary E. Porter, Bradley K. Yoder, Nicolas F. Berbari

**Affiliations:** 1 Department of Cell, Developmental and Integrative Biology, University of Alabama at Birmingham, Birmingham, Alabama, United States of America; 2 Genetics, Cell Biology, and Development, University of Minnesota Medical School, Minneapolis, Minnesota, United States of America; 3 Cell and Developmental Biology, Vanderbilt University, Nashville, Tennessee, United States of America; 4 Division of Nephrology, Department of Medicine, Boston Children’s Hospital, Harvard Medical School, Boston, Massachusetts, United States of America; 5 Department of Medicine, University of Alabama at Birmingham, Birmingham, Alabama, United States of America; 6 Cystic Fibrosis Research Center, University of Alabama at Birmingham, Birmingham, Alabama, United States of America; 7 INSERM, U-983, Necker Hospital, Paris, France; 8 Paris Descartes University, Sorbonne Paris Cité, Imagine Institute, Paris, France; 9 Institut National de la Santé et de la Recherche Médicale (INSERM) Unité Mixte de Recherche (UMR) 1163, Laboratory of Hereditary Kidney Diseases, Paris, France; 10 Assistance Publique-Hôpitaux de Paris, Department of Genetics, Necker Hospital, Paris, France; 11 Department of Medicine, UNC School of Medicine, Marisco Lung Institute, Chapel Hill, North Carolina, United States of America; 12 Department of Pediatrics, UNC School of Medicine, Marisco Lung Institute, Chapel Hill, North Carolina, United States of America; 13 Department of Pathology and Laboratory Medicine, UNC School of Medicine, Marisco Lung Institute, Chapel Hill, North Carolina, United States of America; 14 Department of Genetics, University of Alabama at Birmingham, Birmingham, Alabama, United States of America; 15 Nephrology Division, Massachusetts General Hospital, Charlestown, Massachusetts, United States of America; 16 Department of Pharmacology and Toxicology, University of Alabama at Birmingham, Birmingham, Alabama, United States of America; 17 Department of Biology, Indiana University-Purdue University Indianapolis, Indianapolis, Indiana, United States of America; Washington University School of Medicine, UNITED STATES

## Abstract

Ciliopathies are genetic disorders arising from dysfunction of microtubule-based cellular appendages called cilia. Different cilia types possess distinct stereotypic microtubule doublet arrangements with non-motile or ‘primary’ cilia having a 9+0 and motile cilia have a 9+2 array of microtubule doublets. Primary cilia are critical sensory and signaling centers needed for normal mammalian development. Defects in their structure/function result in a spectrum of clinical and developmental pathologies including abnormal neural tube and limb patterning. Altered patterning phenotypes in the limb and neural tube are due to perturbations in the hedgehog (Hh) signaling pathway. Motile cilia are important in fluid movement and defects in motility result in chronic respiratory infections, altered left-right asymmetry, and infertility. These features are the hallmarks of Primary Ciliary Dyskinesia (PCD, OMIM 244400). While mutations in several genes are associated with PCD in patients and animal models, the genetic lesion in many cases is unknown. We assessed the *in vivo* functions of Growth Arrest Specific 8 (GAS8). GAS8 shares strong sequence similarity with the *Chlamydomonas* Nexin-Dynein Regulatory Complex (NDRC) protein 4 (DRC4) where it is needed for proper flagella motility. In mammalian cells, the GAS8 protein localizes not only to the microtubule axoneme of motile cilia, but also to the base of non-motile cilia. Gas8 was recently implicated in the Hh signaling pathway as a regulator of Smoothened trafficking into the cilium. Here, we generate the first mouse with a Gas8 mutation and show that it causes severe PCD phenotypes; however, there were no overt Hh pathway phenotypes. In addition, we identified two human patients with missense variants in Gas8. Rescue experiments in *Chlamydomonas* revealed a subtle defect in swim velocity compared to controls. Further experiments using CRISPR/Cas9 homology driven repair (HDR) to generate one of these human missense variants in mice demonstrated that this allele is likely pathogenic.

## Author Summary

Growth-Arrest Specific 8 (Gas8) is implicated in dual roles at both the primary cilium to regulate hedgehog signaling and in motile cilia to coordinate cilia movement. To investigate these roles *in vivo*, we created a Gas8 genetrap mutant mouse. Though no overt primary cilia phenotypes were evident in the Gas8 genetrap mutant mice, there were severe motility defects and the mice presented with Primary Ciliary Dyskinesia (PCD) like symptoms including *situs inversus* and hydrocephalus. We also identified two potential disease causing *GAS8* missense variants (A391V and E199K) in humans. Utilizing CRISPR/Cas9 we generated a mouse to mimic the A391V allele. When we crossed the Gas8^AV^ mutants with the Gas8^GT^ mutant, the compound Gas8^GT/AV^ heterozygous animals developed mild hydrocephalus. Rescue experiments using *Chlamydomonas* with mutations in the Gas8 homolog revealed only a modest decrease in swim velocity raising the possibility that the E199K allele is not pathogenic.

## Introduction

Primary cilia are solitary and immotile cellular appendages that serve as signaling hubs for pathways such as Hedgehog (Hh) during development [1]. Motile cilia initiate and maintain fluid flow and are critical in the brain for cerebral spinal fluid flow and are necessary for mucus transport in the lungs [2]. During development, motile cilia are responsible for initiating flow at the embryonic node which is critical for setting up left-right asymmetry in the mammalian body [3–5].

While all cilia have common core components such as tubulin and intraflagellar transport proteins, motile cilia possess several accessory structures such as inner dynein arms (IDAs), outer dynein arms (ODAs), radial spokes, and the nexin-dynein regulatory complex (N-DRC). In *Chlamydomonas reinhardtii*, data indicate that the N-DRC functions to link the A microtubule of one doublet with the B microtubule of the adjacent doublet. It coordinates the activities of the outer and inner dynein arms to regulate flagellar beat frequency and waveform [6,7]. Studies in *Chlamydomonas* have led to the identification of several N-DRC proteins many of which appear to be conserved in mammals [8,9]. As in *Chlamydomonas*, mutations in putative mammalian N-DRC proteins CCDC164 (DRC1), CCDC65 (DRC2), and most recently, GAS8 (DRC4) are correlated with defects in ciliary motility [10–13].

The human homolog of Gas8 was originally identified in human breast cancer and referred to as Growth Arrest Specific 11 (*GAS11*) [14]. This gene shares 56% protein identity to an N-DRC component in *Chlamydomonas* known as DRC4, the protein product of the paralyzed flagella 2 (*PF2*) gene [15]. Loss of *PF2* (DRC4) in *Chlamydomonas* leads to loss of IDAs and the majority of the N-DRC (N-DRC proteins DRC3-7) visible by transmission electron microscopy (TEM) and results in a slower forward swimming velocity and defective waveform [6,16,17]. The function of Gas8 in the mammalian N-DRC remains poorly understood.

Gas8 localizes to the axoneme of motile cilia and also to the base of primary cilia in vertebrate cells [18]. This led us to question if Gas8 serves as an N-DRC component in motile cilia and whether it has a separate role in non-motile primary cilia. This possibility is supported by data from knockdown studies of Gas8 in NIH3T3 cells showing defects in Hh pathway responses. Expression of truncated versions of Gas8, after knockdown of endogenous Gas8, revealed that the C-terminal region of Gas8 bound to and facilitated the transport of Smoothened into the cilium in response to Hh pathway activation using the Smoothened agonist (SAG) [18,19]. In mammals, cilia are essential for normal regulation of Hh signaling activity with many of the Hh signaling components such as Smoothened, Patched and Gli transcription factors dynamically localizing in primary cilia [20–22].

Primary Ciliary Dyskinesia (PCD, OMIM #244400) is a human disease characterized by abnormal motile cilia. PCD patients exhibit bronchiectasis, infertility, and chronic respiratory infections, and in some cases can present with hydrocephalus. A subset of PCD patients will also have a reversal of their left-right body axis that includes *situs inversus totalis* which is referred to as Kartagener syndrome [23]. PCD patients often have changes in cilia axonemal ultrastructure that include defects in the inner or outer dynein arms, central complex, radial spokes, and the N-DRC [24–28]. These structural defects alter ciliary beat frequency (CBF), ciliary waveform, and cilia orientation. Recent data indicate that mutations in the putative mammalian N-DRC components CCDC164, CCDC65, and Gas8 correlate with the clinical presentation of PCD. Mutations in these genes lead to dyskinetic cilia with subtle changes in cilia ultrastructure pointing to an importance for these components in ciliary motility. Other proteins such as CCDC39 and CCDC40 are responsible for the assembly and attachment of the IDAs and N-DRC in motile cilia. The absence of these proteins results in severe motility defects [10,11,29–31].

In this study, we investigate a role for Gas8 in both primary and motile cilia *in vivo*. For this we generated a Gas8 genetrap mutant mouse. Gas8 mutants present with severe hydrocephalus and cilia motility defects on both the ependyma and trachea, as well as a *situs inversus* phenotype. Given the role for Gas8 in cilia motility and recent data suggesting it is a PCD causing allele, we screened human PCD patients for *GAS8* mutations and identified two independent missense variants. The potential pathogenicity of these alleles was tested by rescue experiments in *Chlamydomonas PF2* mutants and by generating a mouse model for one of the variants. In contrast to the PCD phenotypes, we did not observe Hh associated defects in any of the mutant mice or cell lines derived from them or other phenotypes typically associated with defects in primary cilia function. These results suggest that *GAS8* plays a highly conserved role in ciliary motility and mutations in Gas8 are associated with human disease through their impact on motile cilia.

## Results

### Generation of Gas8 mutant mice and phenotype description

**Fig 1:**
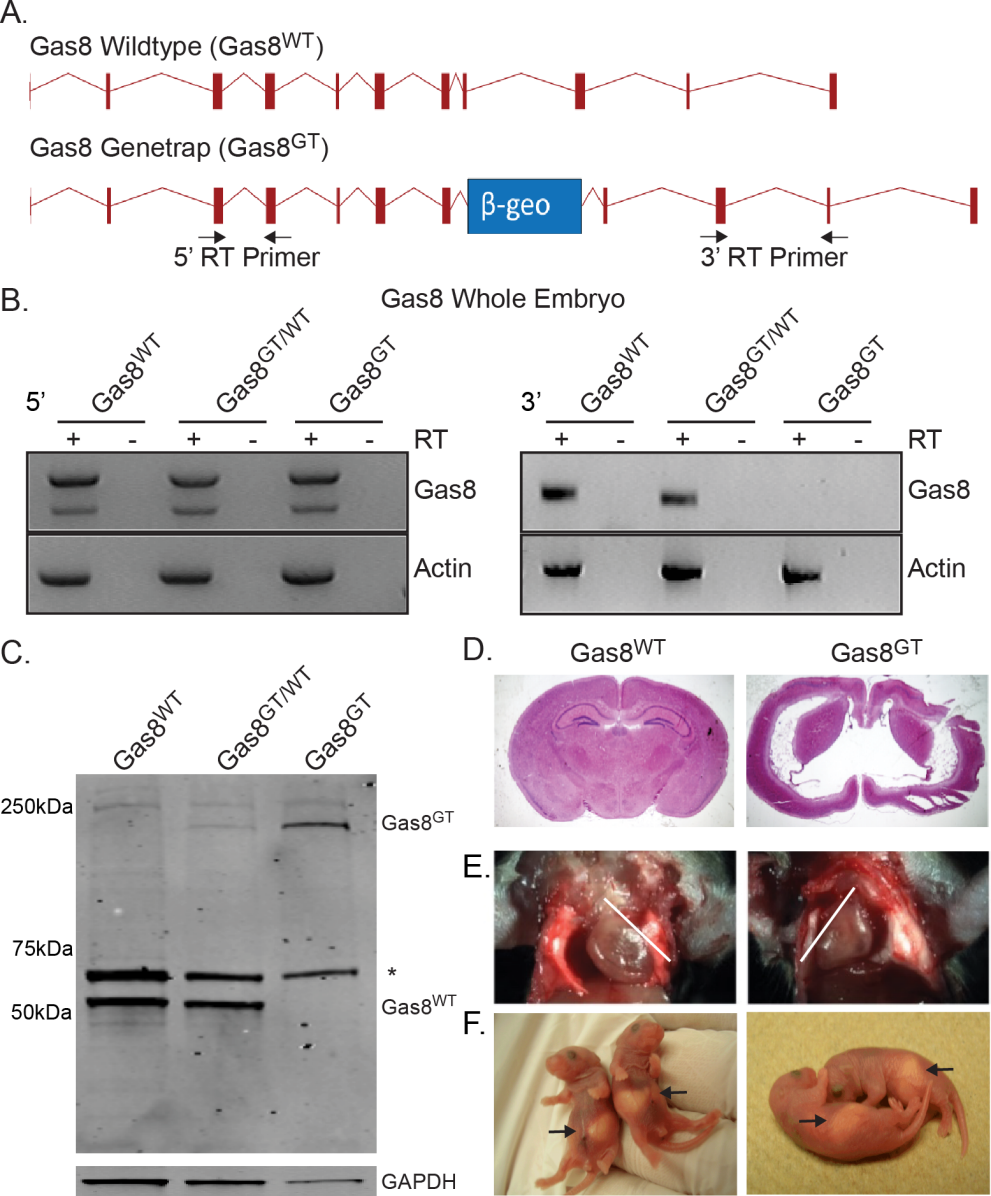
Generation of mutant Gas8^GT^ mice and phenotype description. (A) Schematic of the wildtype Gas8 allele (Gas8^WT^) and the Gas8 genetrap allele (Gas8^GT^). The relative position of the β-geo cassette is indicated by the blue box. Arrows indicate the primers used for RT-PCR analysis. (B) RT-PCR expression analysis of Gas8 transcript in Gas8^WT^, Gas8^GT/WT^, Gas8^GT^ whole embryos shows the presence of the 5’ end of Gas8 transcript (left panels) and absence of the 3’ end (right panels). Actin served as a positive template control in all samples. Reactions with reverse transcriptase are indicated (+) and negative RT controls (-). (C) Western blot for Gas8 protein on Gas8^WT^, Gas8^GT/WT^, and Gas8^GT^ trachea. Wildtype Gas8 is located at 57 kDa (Gas8^WT^) while the genetrap allele is at approximately 230kDa (Gas8^GT^). * denotes a spurious band recognized by polyclonal antibody. GAPDH was used as a loading control. (D) Nissl stained coronal section of P21 Gas8^WT^ and Gas8^GT^ brain. (E) Gas8^GT^ mice display *situs inversus* as noted by the reversed direction of the heart apex (white lines indicate heart axis) and (F) the stomach location in P2 pups (arrow).

A β-geo cassette containing the β-galactosidase enzyme, a neomycin resistance cassette, an N-terminal splice acceptor and poly-A tail was inserted in intron 7 of the Gas8 mouse allele (Fig 1A, herein referred to as Gas8^GT^). RT-PCR analysis using primers located before the genetrap insertion indicates that the 5’ end of the transcript is generated (Fig 1B, left). In contrast, primers located 3’ to the insertion failed to detect any Gas8 mRNA (Fig 1B, right).

Western blot analysis shows a product of expected size (57kDa) in wildtype and heterozygous Gas8 mice. This product is absent in homozygous mutants (Fig 1C). Additionally, the Gas8::β-geo fusion protein is detected (approx. 230kDa) in heterozygous and homozygous mutants indicating that the genetrap allele is being transcribed and translated. Loss of Gas8 led to lethality at approximately postnatal day 14 (P14) with few living to P21. All mutants presented with severe hydrocephalus (Fig 1D). Gas8^GT^ mutant mice also presented with *situs inversus* at a rate of 36% (6 of 16 mutants) in live births based on position of the heart and stomach (Fig 1E and 1F). Both the hydrocephalic and *situs inversus* phenotypes suggested a defect in the function of motile cilia.

### Loss of Gas8 does not result in defective Hh signaling

**Fig 2:**
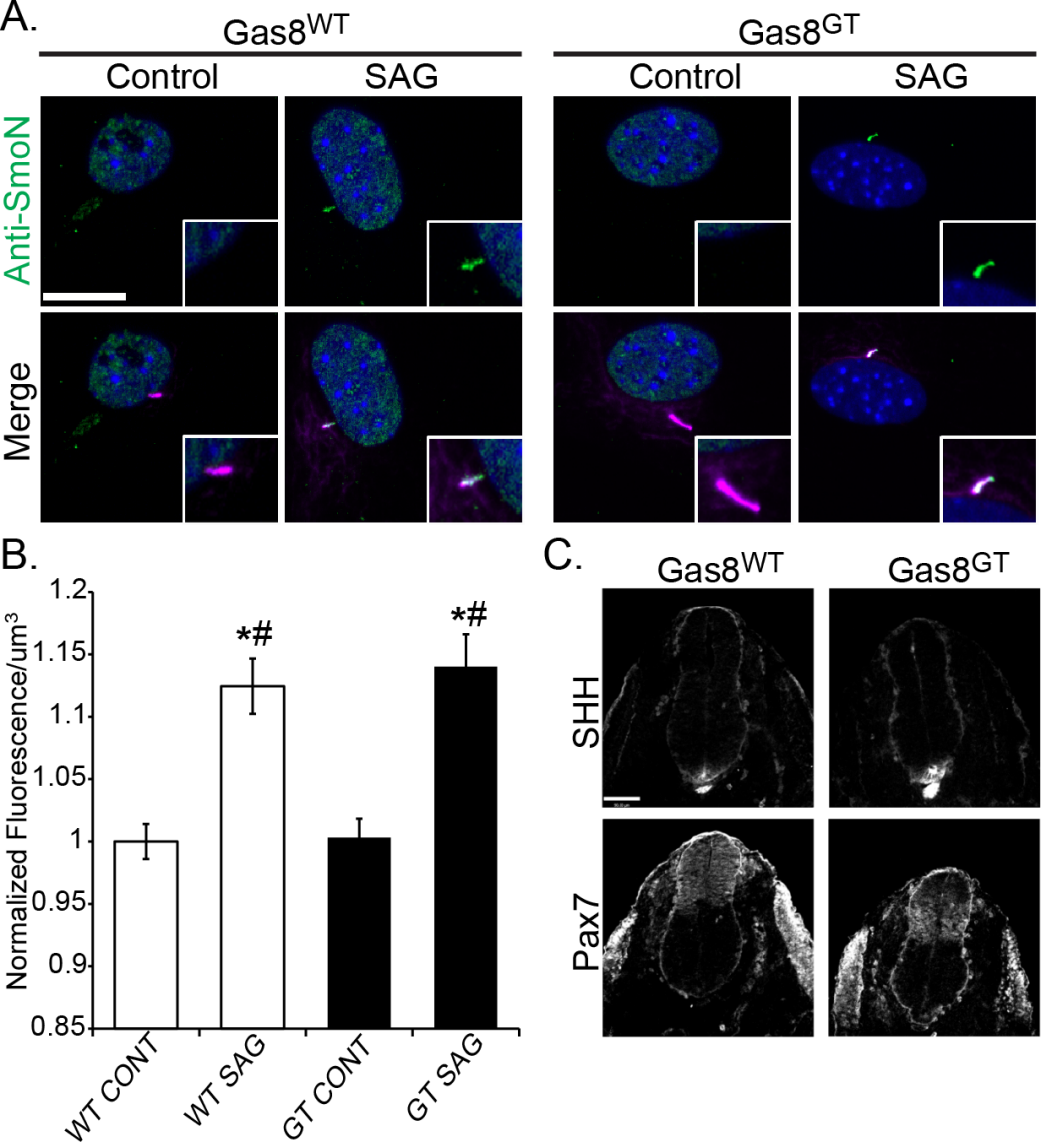
Gas8 mutant embryos have no overt Hh phenotypes. (A) Vehicle (Control) and drug treated (SAG) Gas8^WT^ and Gas8^GT^ mutant MEFs stained for Smo (green) and acetylated tubulin (purple). Scale bar is 15μm. (B) Quantification of Smo translocation into the cilium. All bars are normalized to WT CONT. (* = significantly different than WT CONT, # = Significant different than GT CONT p<0.05). (C) Gas8^WT^ neural tube sections and Gas8^GT^ sections. The Pax7 and Sonic hedgehog (SHH) domains in the notochord, floor plate and neural tube all appear similar between Gas8^GT^ and Gas8^WT^ littermates. Scale is 90μm.

Based on a previous study reporting Gas8 as a positive effector of Hh signaling in mammals, we anticipated Gas8^GT^ mice would present with phenotypes related to Hh signaling defects, especially since this mutation would lack the putative Smo binding domain (amino acids 386–478). However, we did not observe any hedgehog-associated phenotypes in limb patterning or neural tube formation. To further test a role for Gas8 in the Hh pathway, we isolated Mouse Embryonic Fibroblasts (MEFs) from Gas8^WT^ and Gas8^GT^ mutant mice and treated them with 150nM Smoothened agonist (SAG). The MEFs were then immunolabeled for Smo and acetylated tubulin to analyze differences in Smo trafficking into the cilium (Fig 2A). In contrast to the outcome of the knockdown studies, there was no difference between the amounts of Smo present in the cilia of Gas8^GT^ mutants when compared to Gas8^WT^ cilia (Fig 2B). These data indicate that a least in the Gas8^GT^ mutants, Gas8 is not an essential factor involved in regulating Smo cilia trafficking. Similarly, none of the Gas8^GT^ mutants exhibited defects in dorsal ventral patterning of the neural tube typical of altered Hh signaling (Fig 2C).

### Gas8^GT^ mice present with dyskinetic cilia and subtle cilia ultrastructural defects

**Fig 3:**
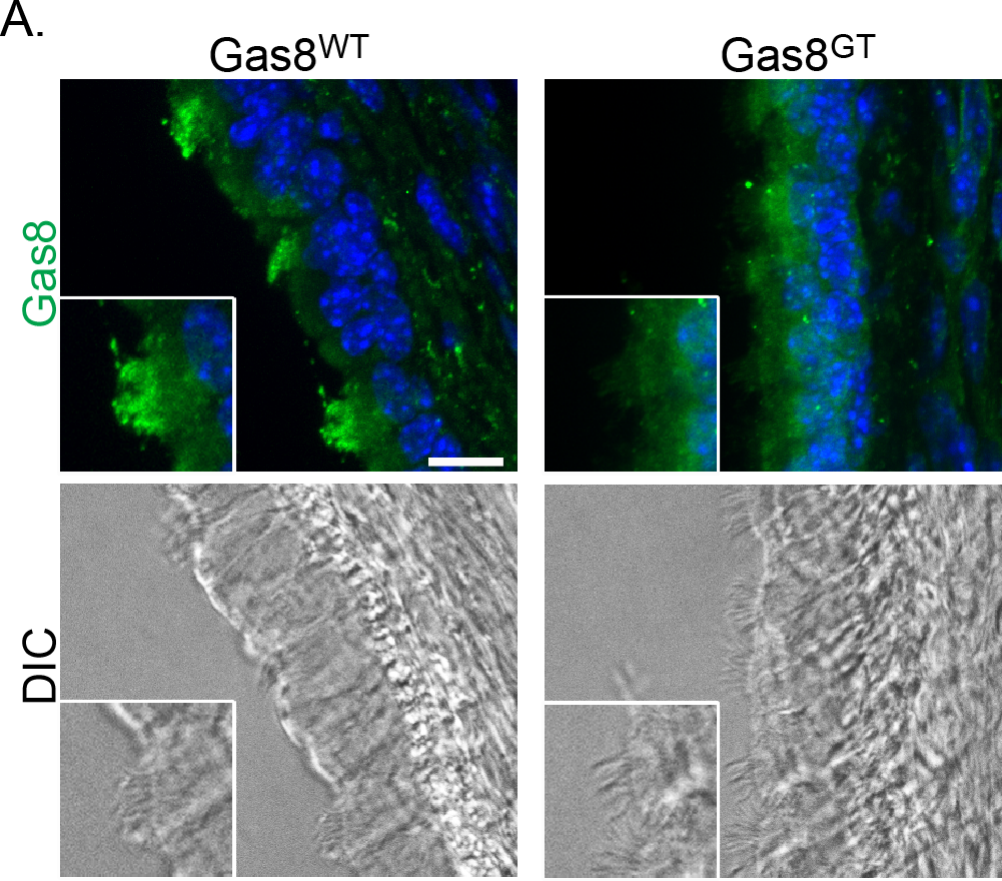
Gas8 localizes to cilia. Gas8 immunofluorescence staining on Gas8^WT^ and Gas8^GT^ p21 trachea (Scale Bar = 10μm). Gas8 is present in wild-type axonemes but absent from mutant axonemes. DIC images are shown to visualize the cilia on the tracheal epithelium.

**Fig 4:**
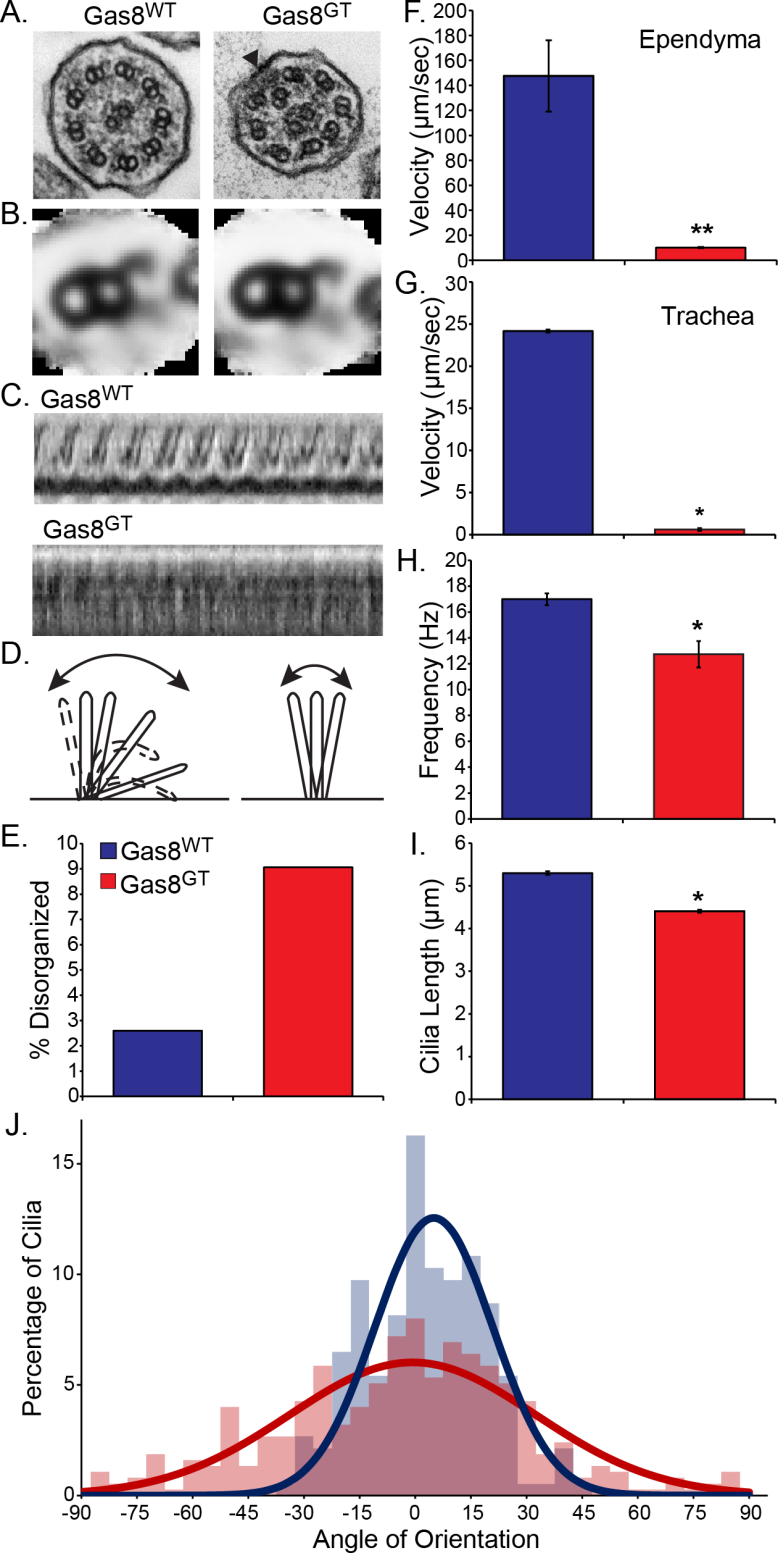
Gas8^GT^ mice present with cilia motility phenotypes. (A) TEM of Gas8^WT^ and Gas8^GT^ tracheal cilia showing disorganized microtubule doublets in mutants. (B) TEM average of tracheal cilia microtubule doublet showing no major structural defects (Gas8^WT^ N = 202 doublets from 1 trachea, Gas8^GT^ N = 202 doublets from 1 trachea). (C) Representative kymograph measurement of ciliary waveform in Gas8^WT^ and Gas8^GT^ tracheal cilia. (D) Schematic depicting waveform defects observed in Gas8^GT^ cilia. (E) Percentage of cilia with disorganized doublets in wildtype and mutant trachea (Gas8^WT^ N = 77 cilia from 1 trachea, Gas8^GT^ N = 287 cilia from 1 trachea). (F and G) Fluorescent bead tracking of Gas8 mice in ependyma and trachea epithelium respectively. Bead flow is significantly impaired in Gas8^GT^ versus Gas8^WT^ Ependyma: Gas8^WT^ = 147.5μm/sec, Gas8^GT^ = 10.2μm/sec (Ependyma: Gas8^WT^ N = 4 brains, Gas8^GT^ N = 4 brains). Trachea: Gas8^WT^ = 24.15μm/sec, Gas8^GT^ = 0.62μm/sec. (Trachea: Gas8^WT^ N = 3 trachea, Gas8^GT^ N = 3 trachea). (H) Tracheal cilia beat frequency (CBF) captured by DIC and analyzed using fast Fourier transform test. Gas8^WT^ CBF = 17.0Hz and Gas8^GT^ CBF = 12.7Hz (Gas8^WT^ N = 108 cilia from 4 trachea, Gas8^GT^ N = 46 cilia from 3 trachea). (I) Cilia length is decreased in Gas8^GT^ mutants as measured from DIC images (p<0.01, Gas8^WT^ N = 321 cilia from 3 trachea, Gas8^GT^ N = 343 cilia from 3 trachea). (J) Cilia orientation is significantly altered in Gas8^GT^ mice (p = 0.025) (Gas8^WT^ N = 184 cilia from 1 trachea, Gas8^GT^ N = 375 cilia from 1 trachea). * = p<0.01, ** = p<0.001

To investigate the hydrocephalus phenotype, cilia morphology, ultrastructure and motility on ependymal and tracheal cells was assessed. DIC analysis and immunofluorescence staining of trachea indicate motile cilia are present on the epithelium, but the Gas8^GT^ protein fails to localize to these cilia (Fig 3). We counted cilia from trachea TEMs for broken doublet rings and found that about 9% of cilia from Gas8^GT^ mutants showed disorganization of the arrangement of the microtubule doublets (Fig 4A arrowhead and 4E). To analyze ultrastructure within the doublets, we averaged 202 doublets of both genotypes to reduce variability due to random sectioning of the 96nm repeat of the microtubule doublet. We did not observe any major structural differences in the inner or outer dynein arms (Fig 4B). However, high speed video and Fourier transformation analysis revealed that cilia are largely static with only a few moving (Fig 4C and 4D, S1 Movie and S2 Movie). Those cilia that did moved were dyskinetic, resulting in an inability of cilia to propel fluid as seen by tracking of fluorescent beads added to either brain ventricle or trachea preparations (Fig 4F and 4G). Beat frequency of cilia that remained motile in Gas8^GT^ mutants was modestly decreased from 17.0Hz in Gas8^WT^ to 12.7Hz in Gas8^GT^ (Fig 4H). Cilia length is also affected in Gas8^GT^ mice, with Gas8^GT^ motile cilia measuring 0.9μm shorter than Gas8^WT^ motile cilia (Gas8^WT^ 5.3μm and Gas8^GT^ 4.4μm) (Fig 4I). Cilia orientation in Gas8^GT^ tracheas is also more randomized than in Gas8^WT^ controls (Fig 4J). These phenotypes observed in the motile cilia of Gas8^GT^ mutant mice are similar to those observed in PCD patients and animal models.

### GAS8 is a disease causing gene in humans

**Fig 5:**
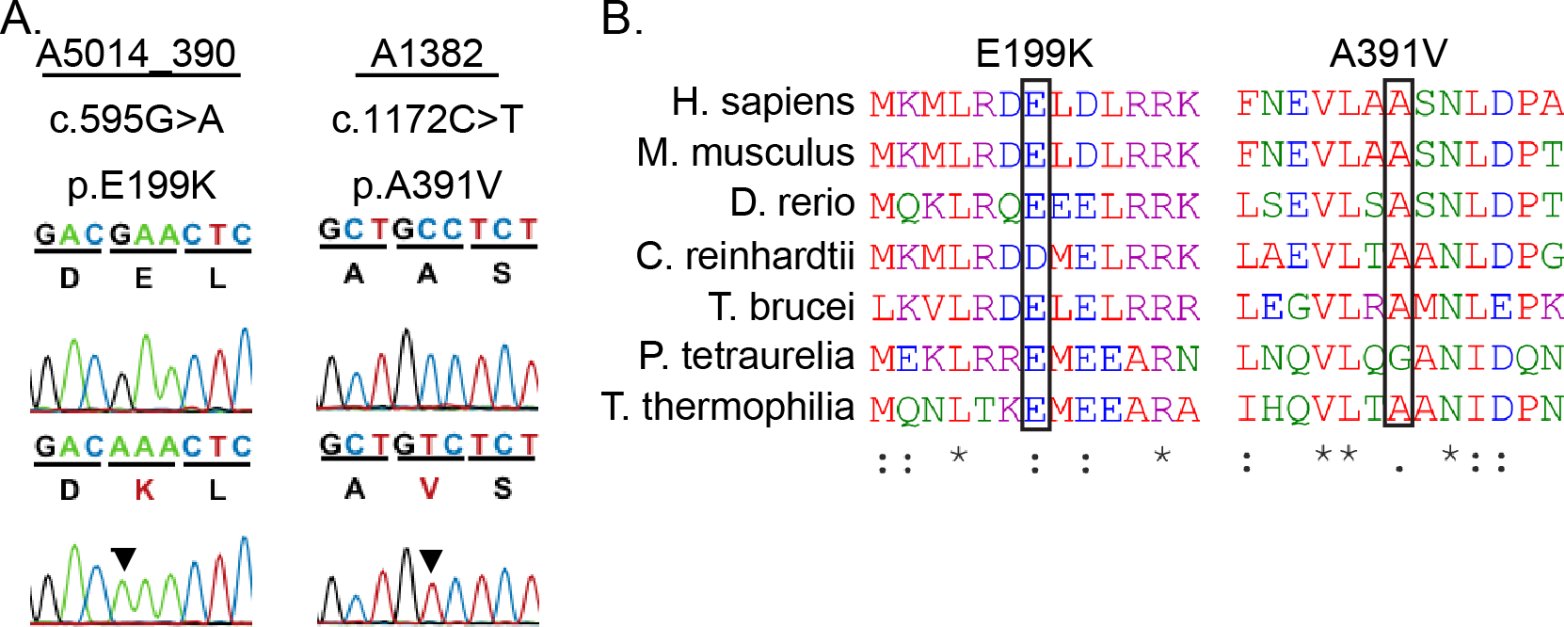
PCD patient missense mutations in highly conserved regions of Gas8. (A) Sanger Sequence trace and amino acid sequence of human mutations. (B) ClustalOmega amino acid alignment indicates the high conservation of the missense mutant residues found in patients which are indicated by black boxes.

The phenotypes in the Gas8^GT^ mutants led us to evaluate whether mutations in *GAS8* are associated with PCD in humans. We identified two independent missense variants, c.595G>A E199K and c.1172C>T A391V, in human patients through a previously published screen (Fig 5A) [32]. The E119K patient is of Latino decent and presented with heterotaxy. Unaffected parents of the patient are heterozygotes, and an unaffected female sibling is a homozygote. This allele appears at a frequency of 11% in Latino populations (87 homozygotes and 1279 heterozygotes in a total of 11564 alleles sequenced according to ExAC). The prevalence of this allele in the Latino community makes it unlikely to be associated with disease. The A391V patient met the diagnostic criteria for PCD. This allele is infrequent, occurring only 3 times heterozygously and 0 times homozygously in 84864 alleles sequenced according to ExAC. Both variants affect highly conserved regions across multiple species (Fig 5B). We utilized the PolyPhen-2 program to predict the pathogenicity of these alleles. The A391V allele had a PolyPhen-2 score of 0.762 suggesting that it is a potentially damaging mutation while the E199K allele had a score of only 0.082, suggesting that this is a benign mutation. Given the low allele frequency, PolyPhen-2 score, and the confirmation of the PCD diagnosis in the patient carrying the A391V variant, we chose to test potential pathogenicity of this allele in mice.

**Fig 6:**
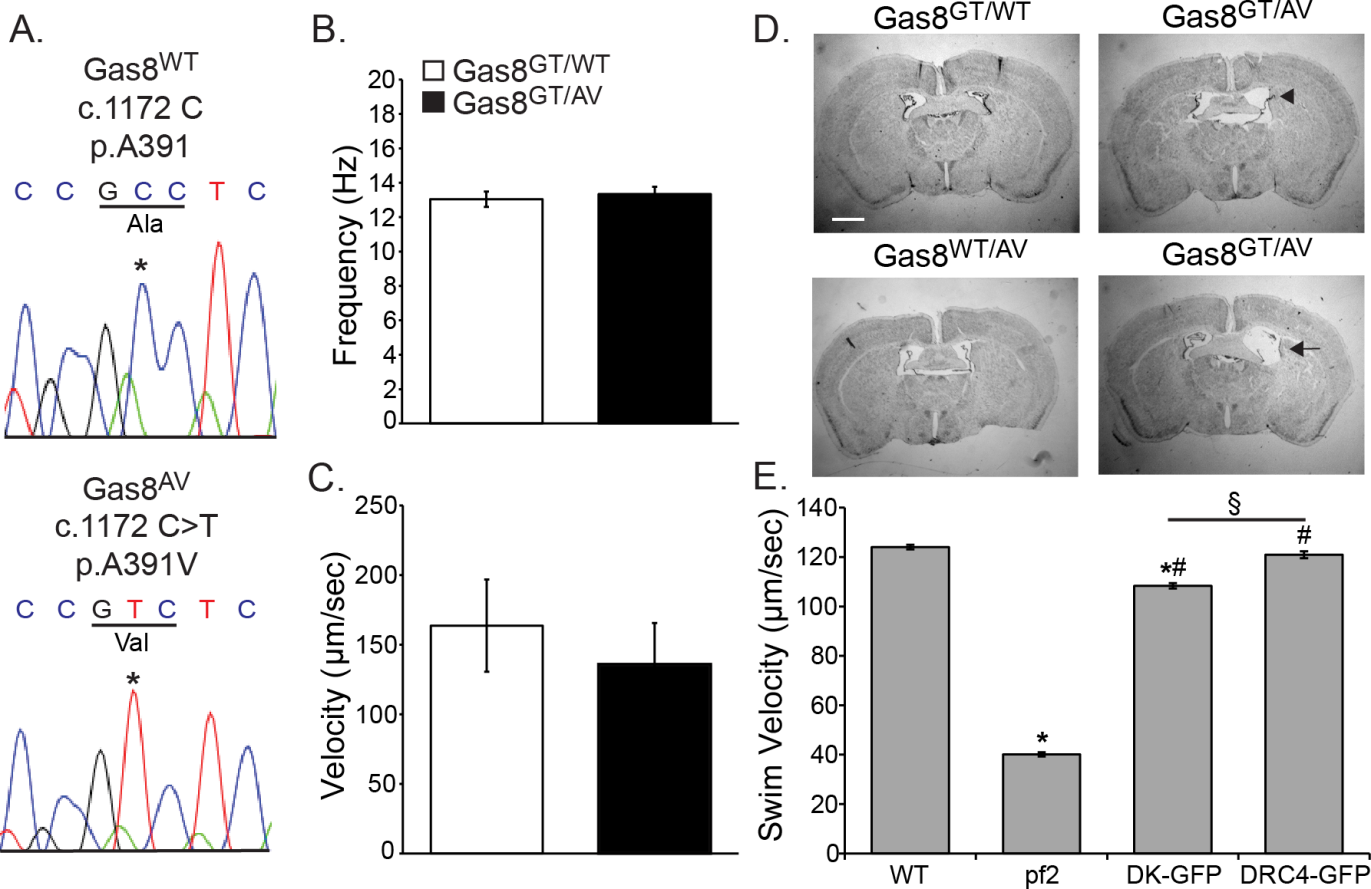
A391V is a potential pathogenic allele. (A) Sanger Sequence confirmation of the 1172 C>T point mutation in Gas8^AV^ mice reproducing the A391V missense mutation found in the human patient. (B) Ciliary beat frequency analysis on tracheal cilia of Gas8^GT/WT^ and Gas8^GT/AV^ mice shows no difference between controls and compound heterozygotes (n = 86 points from 3 trachea for Gas8^GT/WT^ (13.04 Hz), n = 76 points from 3 tracheas for Gas8^GT/AV^ (13.34 Hz)). (C) Tracking of red fluorescent latex beads added to lateral ventricles shows a trending but not significant decrease in ability of Gas8^GT/AV^ cilia to move fluid. (n = 3 for Gas8^GT/WT^ (163.7μm/sec), n = 2 for Gas8^GT/AV^ (135.9μm/sec)). (D) Nissl stained brains of 10 week old Gas8^GT/WT^, Gas8^WT/AV^, and Gas8^GT/AV^ mice. Mild to moderate hydrocephalus is present in the Gas8^GT/AV^ brains. Scale is 1mm (Arrowhead indicates mild, arrow indicates moderate) (n = 4). (E) Swim speed quantification of rescue of DRC4-D198K construct in *pf2* deficient *Chlamydomonas*. “*pf2*” denotes *pf2* deficient *Chlamydomonas*, “DK-GFP” denotes *pf2* deficient *Chlamydomonas* expressing the DRC4-D198K-GFP construct, “DRC4-GFP” denotes *pf2* deficient *Chlamydomonas* expressing the DRC4-GFP wild-type construct. * = significant difference from WT (p<0.05), # = significant difference from *pf2* (p<0.05), § = significant difference between DK-GFP and DRC4-GFP (p<0.05) (n = 390 for WT (123.9μm/sec), n = 271 for *pf2* (40.1μm/sec), n = 180 for DK-GFP (108.3μm/sec), n = 299 for DRC4-GFP (120.9μm/sec)).

To further assess the potential pathogenicity of the human allele, we created a mouse harboring the A391V mutation via homology driven repair with CRISPR/Cas9 technology. Sequencing confirmed the presence of the c.1172 C>T mutation resulting in an A391V amino acid change (Fig 6A). We crossed Gas8^AV^ mice onto the Gas8^GT^ background to create compound heterozygous (Gas8^GT/AV^) mice. To determine the impact on motile cilia and test possible cause of the hydrocephalus, we took brains from 6 week old mice and analyzed cilia beat and the ability of motile cilia to move fluid. While there were no differences in beat frequency, bead flow analysis shows a modest decrease in the ability of Gas8^GT/AV^ cilia to move fluid compared to Gas8^GT/WT^ cilia (Fig 6B and 6C). Compound heterozygotes develop mild hydrocephalus at approximately 10 weeks of age (Fig 6D) but there were no evident laterality defects. While all the Gas8^GT/AV^ mice analyzed (n = 6) display hydrocephalus at this age, the severity ranged from mild (arrowhead) to moderate (arrow). The phenotype in the Gas8^GT/AV^ mice is not as severe as in the Gas8^GT^ mice and hydrocephalus was not present in any (n = 2) of the Gas8^GT/WT^ or (n = 2) of the Gas8^WT/AV^ mice analyzed.

We chose to generate the A391V mouse model because of the PCD symptoms of the patient but given the lack of full PCD symptoms in the E199K patient, we decided to test first whether or not the E199K is pathogenic in *Chlamydomonas* before proceeding to a potential mammalian model. Alignment of GAS8 and the *Chlamydomonas* orthologue DRC4 revealed that E199 in GAS8 aligns with D198 in DRC4 (Fig 5B). To better understand the mechanisms underlying these defects, we generated strains expressing the *Chlamydomonas* equivalent (D198K) of the human E199K alleles in a null mutant background (*pf2*). Interestingly, transformation with *DRC4-DK-GFP* rescued the severe motility defects seen in the *pf2* null mutant, but measurements of forward swimming velocities revealed a subtle defect in the swimming phenotype of the rescued strains (Fig 6E). Furthermore, the DRC4-DK-GFP protein is assembled at wild-type levels in the flagellar axonemes of *Chlamydomonas*, as assayed by western blot (S1 Fig). These observations show that the D198K DRC4 mutant protein is properly localized in the axoneme and may not correspond to a pathogenic allele.

## Discussion

Defects involving cilia motility cause severe phenotypes in humans including infertility, hydrocephalus, respiratory defects, and reversal of left-right asymmetry. Much of our understanding about cilia motility has come from studies in organisms such as *Chlamydomonas*. These studies and how defects in cilia motility cause disease are now being extended into mammalian systems. Recently GAS8 was implicated as a cause for Primary Ciliary Dyskinesia (PCD) as well as a positive effector of Smoothened transport into cilia during Hh pathway activation [12,13,19].

To further evaluate the connection between Gas8 and PCD in mammals, we generated a mouse with a β-geo cassette inserted in intron 7 of the Gas8 gene. Insertion of the β-geo genetrap cassette effectively eliminated the presence of wildtype transcript and protein in mutants as verified by RT-PCR and western blot analysis. Though the Gas8^GT^ mutant allele is translated into a large fusion protein between the N-terminal portion of Gas8 and β-geo, it does not localize to motile cilia. Gas8^GT^ mutant mice present with hydrocephalus starting at postnatal day 5 (P5) that becomes more pronounced as the mice mature and eventually leads to mortality between P14-P21. Development of hydrocephalus is associated with severe impairment of cilia motility on ependymal cells lining the ventricles of the brain.

Previous studies using image average procedures to analyze flagella ultrastructure in *Chlamydomonas* showed that strains with mutations in PF2/DRC4, the Gas8 homolog, were associated with the loss of the majority of the N-DRC complex along with a subset of the IDAs [6,16,17,33]. In contrast to the *Chlamydomonas* results, the N-DRC and IDA do not appear to be overtly affected in Gas8 mutant mice based on standard thin-section TEM analysis. However, loss of Gas8 does effect microtubule organization, as indicated by a higher percentage of cilia with disorganized microtubule doublets in Gas8^GT^ mutant mice when compared to Gas8^WT^ mice. Altered cilia microtubules were recently also recently observed in human Gas8 patients [12,13]. Together these data suggest that defects in the mammalian N-DRC may not always be detectable using traditional TEM averaging of cross-sections. The inability to observe ultrastructural defects in human PCD patients could be attributed to having only one N-DRC per 96nm repeat. Future studies using better imaging approaches such as cryo-electron tomography and image averaging of longitudinal sections to assess the human N-DRC will likely continue to reveal structural and functional differences similar to those described for the radial spokes by Lin, *et al* 2014.

Most Gas8^GT^ mutant cilia failed to move, however those that were observed moving displayed a modest decrease in beat frequency. The most distinguishable phenotype observed in the cilia that moved was a very rigid and short wave pattern. This pattern has also been observed in other cilia motility mutants thought to affect the NDRC [10–12]. These changes in waveform and the lack of overall motility result in the defective fluid flow observed in these mice. Previous data show a complex relationship between planar cell polarity (PCP) and fluid flow in establishing motile cilia orientation [34,35]. Gas8^GT^ cilia show a more random distribution of cilia orientation than their Gas8^WT^ counterparts supporting the necessity of proper fluid flow in establishing cilia orientation.

Variants in Gas8 were recently identified in human PCD patients. These mutations resulted in a similar, albeit not significant, decrease in beat frequency along with an abnormally rigid ciliary waveform [12,13]. This motility phenotype is similar to our observation in the mutant mice. Here we identified an additional independent missense mutation, c.1172C>T A391V, in PCD patients as well as a variant c.595G>A E199K that appears to have minimal effect on cilia motility. The A391V mutations lies in close proximity to the other published mutants, C309*, A334*, and G357* suggesting that this area is critical for GAS8 function. Similarly, the genetrap cassette in the Gas8^GT^ allele was inserted in close proximity (K337) to the A334* mutation. The E199K mutation also affects a highly conserved region within Gas8 that is proposed to be a Microtubule Association Domain (GMAD) [36].

To test pathogenicity of the A391V allele, we used CRISPR/CAS9 homology driven repair (HDR) to generate a mouse line mimicking the human mutation. Mice compound heterozygous for the Gas8^GT^ and Gas8^AV^ mutations develop age dependent, mild hydrocephalus, but did not present with situs defects (n = 6 Gas8^GT/AV^ mice). The phenotype was associated with a reduced ability of ependymal cilia to move fluid. Interestingly, beat frequency was not significantly altered from that of controls, suggesting that the defect lies within a subtle waveform difference or in cilia orientation. These data suggest that the A391V allele is pathogenic though more in-depth analysis of ciliary defects will be necessary to determine the precise mechanism. Data from the *Chlamydomonas* rescue experiments suggest that the E199K allele may have very subtle effects on motility. The D198K rescued strain in *Chlamydomonas* showed a small but statistically significant reduction in forward swim velocity of approximately 10 percent. While statistically significant, additional work is needed to determine whether such small changes might impact ciliary motility and have pathogenic consequences in different tissues and different organisms. As this variant is commonly found in Latino populations, it seems more likely that this variant is a benign polymorphism.

Gas8 was previously implicated as a modulator of the Hh pathway. *In vitro* data indicated that the C-terminal region of Gas8 binds to Smoothened (Smo) and acts at the base of primary cilia as a regulator of Smo entry into the cilium following Hh pathway activation [19]. These data showed that in the absence of Gas8, Smo accumulation in the cilium is abrogated and that it cannot activate the Gli transcription factors and turn on downstream genes. Based on these *in vitro* findings, we expected to see Hh defects in our mutant mice. However, the Gas8 mutants survive to birth and have normal digit number and patterning as well as normal neural tube dorsal ventral patterning. Furthermore, there were no significant differences in Smo accumulation in cilia between Gas8^WT^ and Gas8^GT^ MEFs after SAG stimulation, suggesting that in this mutant model, Gas8 does not act as a regulator for Smo entry. The role that Gas8 plays at the base of primary cilia remains uncertain; however, we do not see any other pathologies that would suggest there is a defect in primary cilia such as cystic kidney disease.

The data presented here solidify *GAS8* as a disease causing gene in humans and elucidate the mechanisms by which loss of Gas8 causes disease. We identified new independent, homozygous missense mutations and used model systems to test the pathogenicity of the alleles. Importantly, these results suggest the A391V allele is pathogenic while the E199K variant is not. Our results demonstrate the importance of testing the potential pathogenicity of human alleles in easily amenable model systems such as *Chlamydomonas* and further reveal the ease with which CRISPR/Cas9 has now made it possible to conduct similar tests in mouse models.

## Materials and Methods

### Mice

The Gas8 mutant mouse line was generated using embryonic stem cell line CH0760 (BayGenomics) in which a β-galactosidase neomycin resistance fusion cassette was inserted into intron 7 of Gas8. The insertion site was confirmed by genomic PCR and sequence analysis. PCR primers for genotyping were designed based on the insertion site and are as follows 5’-GGGACAAGCAGATTCTGGTC-3’, 5’-CAGGGTTACACACAGAGAAACC-3’, and 5’-CCGCAAACTCCTATTTCTG-3’. The Gas8^GT^ embryonic stem cells were from the 129P2/OlaHsd genetic background and were injected into C57BL/6 blastocysts using standard procedures. Chimeras were bred with albino C57BL/6 females and germline transmission was confirmed by coat color and subsequent PCR genotyping.

### Ethics statement

All experimental procedures were approved by the Institutional Animal Care and Use Committee (IACUC) regulations at the University of Alabama at Birmingham under the animal protocol number (130208061).

### RT-PCR

RNA was isolated from Gas8^WT^, Gas8^GT/WT^, and Gas8^GT^ mouse embryonic fibroblasts using Trizol reagent according to the manufacturer’s protocol (cat# 15596–026, Thermo-Fisher Scientific). cDNA was generated using Verso cDNA kit (cat# AB-1453/B, Thermo-Fisher Scientific). 5’ Gas8 RT-PCR was performed using the following primers spanning exons 3 and 4: 5’-GAATCGAAGAATACCACCATC-3’ and 5’-CTGAGAAGATGGCTATGTAG-3’. 3’ Gas8 RT-PCR was performed with primers spanning exons 9 and 10: 5’-CTGGACCCCACAGCATTAAC-3’ and 5’-CTTGATGGTGGTATTCTTCG-3’. Actin control primers: 5’-ATGGGTCAGAAGGACTCCTA-3’ and 5’-GGTGTAAAACGCAGCTCA-3’ were used in all samples.

### Tissue preparation

Animals were anesthetized by a 0.1 ml per 10 g of body weight intraperitoneal injection of 2.5% tribromoethanol (cat# T48402, Sigma–Aldrich), killed by cardiac puncture, and perfused with PBS followed by 4% paraformaldehyde (cat# 19943, Thermo-Fisher Scientific). The brains were further fixed in 4% paraformaldehyde 1h at room temperature followed by successive dehydration through 1 hour alcohol incubations at 30% and 50% and placed finally in 70% overnight. Tissues were further dehydrated through 1 hour alcohol incubations at 80%, 95%, and finally 100%. Tissues were placed in xylenes for 1 hour and then placed in a 50/50 xylenes/paraffin mix for 1 hour at 60°C under vacuum followed by a final paraffin penetration in paraffin at 60°C under vacuum for 1 hour and then paraffin embedded. The brains were sectioned at 10μm and stained with Cresyl Violet stain as previously described [37].

### Immunoblotting

Fresh tracheas were extracted from p21 Gas8^WT^, Gas8^GT/WT^, and Gas8^GT^ mice. Samples were submerged in ice cold RIPA (10mM Tris pH7.5, 150mM NaCl, 1%NP-40, 1% sodium deoxycholate, 0.1% SDS) mixed with one cOmplete Protease Inhibitor tablet (cat# 11 836 170 001, Roche Diagnostics) per 10mL at 300uL per 5mg of tissue. Tissues were sonicated 3x for 10 seconds each. After sonication, tissues were placed on a rotary agitator for 2 hours at 4°C and then spun for 20 minutes at 12,000rpm at 4°C. Supernatant was removed and protein levels were assayed using a BioRad DC protein assay kit (cat# 5000111, Bio-Rad). Approximately 20μg per sample was used for SDS-PAGE with a 12% Tris-Glycine gel (cat# 00252562, Thermo—Fisher Scientific). Proteins were transferred overnight to nitrocellulose. The membrane was blocked for 45 minutes in 5% milk in PBS and incubated with primary antibody in 5% milk in PBS with 0.02% Tween-20 overnight at 4°C. Primary rabbit anti-Gas8/DRC4 antibody was used at 1:20000 [6]. Primary mouse anti-GAPDH was used as a loading control at 1:1000 (cat# ab8245, Abcam, Cambridge UK). Blots were washed 5x for 5 minutes each in 0.02% PBS-Tween-20. Secondary antibody in 5% milk in 0.02% PBS-Tween-20 was added and the blot was incubated for 1 hour at room temperature with the following secondary antibodies: anti-mouse IRDye 800CW (cat# 827–08364, LI-COR, Lincoln NE USA) and anti-rabbit IRDye 680RD (cat# 926–68071, LI-COR). Blots were washed 5x for 5 min each in 0.02% PBS-Tween-20 and then dried. Images were taken on a LI-COR Odyssey CLx imaging system (LI-COR).

### Mouse embryonic fibroblast generation

Mouse embryonic fibroblasts (MEFs) were generated from E14.5 embryos and cultured in DMEM growth medium with High Glucose, 0.05mg/ml Penicillin/Streptomycin, 2mM L-Glutamine, 0.2mM β-mercaptoethanol, and 20% Fetal Bovine Serum (FBS). Prior to immunolabeling, MEFs were cultured in reduced serum medium containing 0.5% FBS for 48 hours to induce primary cilia formation as previously described [38].

### Immunofluorescence

Cells were fixed in 4% paraformaldehyde and permeabilized with 0.1% Triton X-100 in PBS with 2% donkey serum, 0.02% sodium azide and 10 mg/ml bovine serum albumin (BSA). Cells were labeled with anti-acetylated α-tubulin, 1:1000 (cat# T-6793, Sigma-Aldrich), anti-SmoN, 1:1000 (gift from Dr. Matthew Scott, Stanford University). Sections from E10.5 neural tubes were immunolabeled with the following antibodies from Developmental Studies Hybridoma Bank (University of Iowa, Iowa City, IA): anti-ShhN 1:1000 (5E1), anti-FoxA2 1:1000 (74.5a5), anti-Mnr2 1:1000 (81.5C10), anti-Pax7 1:1000 (Pax7), and anti-Msx1+2 1:1000 (4G1) as previously described [38]. Trachea sections were labeled with anti-Gas8/DRC4, 1:2000 [6]. All incubations and washes were carried out in PBS with 2% normal donkey serum, 0.02% sodium azide and 1% BSA. Primary antibody incubations were performed for 16–24 hours at 4°C and secondary antibody incubations were performed for 1 hour at room temperature. Secondary antibodies all from Thermo-Fisher Scientific include the following: Alexa Fluor-594 donkey anti-mouse (cat# A21203), Alexa Fluor-488 donkey anti-mouse (cat# A21202), Alexa Fluor-594 donkey anti-rabbit (cat# A21207), and Alexa Fluor-488 donkey anti-rabbit (cat# A21206). Nuclei were visualized by Hoechst nuclear stain. Coverslips were mounted using Immu-Mount (cat# 9990402, Thermo-Fisher Scientific). Fluorescence imaging was performed using a Nikon TE-2000U inverted microscope (Melville, KY) outfitted with a PerkinElmer UltraVIEW ERS 6FE-US spinning disk laser apparatus (Shelton, CT) and a Hamamatsu C9100.

DIC images of p14 trachea prepared for IF were used for length analysis. Images were captured with a 40x objective (Plan-Fluor, 1.3NA). Length was measured manually by drawing a line from the tip of the cilium to the base using Volocity v6.3.

### Smoothened trafficking assay

Gas8WT and Gas8GT MEFs were grown to confluency on 0.17mm coverslips and serum starved for 48 hours to induce ciliation. Cells were treated with 150nM Smoothened agonist (SAG) (cat#566660, CALBIOCHEM) for 2 hours in low serum media to induce Hedgehog pathway activation and Smoothened translocation. Cells were fixed and stained as described in the immunofluorescence section and imaged by spinning disk confocal. Amount of Smoothened per cilia volume was measured using Volocity v6.3 software.

### Transmission electron microscopy

Postnatal day 14 (P14 mice were anesthetized and perfused with PBS followed by a perfusion of 2% glutaraldehyde in 0.1M cacodylate buffer pH 7.4. Tracheas were extracted and fixed overnight at 4°C in 2% glutaraldehyde in 0.1M cacodylate buffer pH 7.4. Samples were then washed thoroughly four times for 15 minutes each in 0.1M cacodylate Buffer pH 7.4. A post fix in 1% OsO4 in 0.1M cacodylate buffer pH 7.4 was performed. Samples were washed two times for 10 minutes each in 0.1 M cacodylate pH 7.0. Samples were then prepped in 1% tannic acid in 0.1M cacodylate Buffer pH 7.0; 30 minutes followed by 1% NaSO4 in 0.1M cacodylate Buffer pH 7.0; 5 minutes. Dehydrate the samples in 50%, 75%, and 95% at 4°C for 20 minutes each and finally 100% EtOH for 20 minutes; warm to RT°. Dehydrate samples totally with four washes of 100% EtOH 15 minutes each. Infiltrate the sample with Propylene Oxide for 30 minutes. Mix the EMbed 812 according to instructions from EMS and Infiltrate with 25% Embed in propylene oxide for 30 minutes, 50% for 40 minutes, 75% overnight, 100% for four hours, 100% for 1 hour and harden at 60°C. Samples were sliced at 90nm and imaged on a Phillips CM110 Electron Microscope.

TEM averaging of doublets was performed by isolating individual doublets from cilia and importing the doublets into Photoshop CS5. Individual doublets were aligned to a single template doublet and then averaged and flattened.

TEMs were used to determine cilia orientation. Cilia orientation was determined by measuring the angle of central pairs by drawing a line across the central doublets and measuring the angle relative to the image. Each angle was normalized to the average (or most common angle) after setting the average angle to 0°. The frequency of angles in each image was measured and plotted.

### Bead flow analysis

Brains of experimental mice were extracted, sliced in half to expose the ependymal of the lateral ventricles and placed in pre-warmed, pre-oxygenated artificial cerebrospinal fluid (125mM NaCl, 2.5mM KCl, 1.25mM NaH_2_PO_4_, 2mM CaCl_2_, 1mM MgCl_2_, 25mM NaHCO_3_, 25mM Glucose, pH 7.35). Brains were placed on a Zeiss Axioskop microscope and imaged with a 5x objective (Plan-Neofluor, 0.15NA) and a 10x objective (Fluor, 0.5NA) using a Photometrics CoolSnap HQ CCD camera at 30fps. Red fluorescent latex beads (cat# L3530-1mL, Sigma-Aldrich) were diluted 1:100 from stock and 10μL of diluted beads was added to the ventricles. Bead tracking analysis was performed using the MTrack2 plugin in FIJI.

### High speed video microscopy

Mice trachea were dissected out into fresh PBS and cut lengthwise into strips. Trachea were kept in warm media (DMEM F/12, 20% FBS, and Pen-Strep and allowed to adapt for 20 minutes in an environmental chamber (37°C, 45% relative humidity, and 5% CO_2_) before imaging with Differential Interference Contrast (DIC). All high speed video was captured at 240fps using a modified Casio Exilim EX-ZR100 attached to a Nikon TE-200 using a 60x water objective (Plan-Apo WI NA = 1.2). Videos saved as quick time files were then extracted into individual frames using VirtualDub 1.10.4 software and all analysis was performed in ImageJ.

Kymographs were created using Metamorph v6.1. A line was drawn from the tip of the cilium to the base and kymographs were made from the results.

### Generation of Chlamydomonas lines

To make the D198K mutation in *Chlamydomona*s, the DRC4-GFP plasmid [6] was used as template for PCR with the primers 5’-CAGTGCTGTGAGCCTGACG and 5’-AAACCAAAGCACCTTGAGCG to generate a 1483bp product that contains the restriction sites *Bcl*I and *Cla*I flanking the desired mutation site. The PCR product was cloned into pGEM-T-Easy (cat# A1360, Promega Corp) to generate the plasmid pf2-Y1-A. This plasmid was further digested with *Kpn*I and *Spe*I to removed repetitive DNA and subcloned into pBlueScript to generate the plasmid pf2-Y1-B. The D198K mutation was introduced into pf2-Y1-B using the primers 5’-GAAGATGCTGCGAGACaAaATGGAGCTGCGGAGAAAG-3’ and 5’-CTTCTACGACGCTCTGtTtTACCTCGACGCCTCTTTC-3’ and the QuickChange II kit (Agilent Technologies) to generate the plasmid pf2-Y1-C. After sequence verification by Genewiz, the pf2-Y1-C plasmid was digested with *Kpn*I and *Spe*I and subcloned back into pf2-Y1-A to generate the plasmid pf2-Y1-D. The pf2-Y1-D plasmid was digested with *Bcl*I and *Cla*I to release the 1483 bp fragment now carrying the D198K mutation. This fragment was subcloned back into the original DRC4-GFP plasmid by Genewiz. The completed plasmid, DRC4-D198K-GFP, was linearized with *EcoR*I for transformation into the *pf2-4* strain [6]. Transformants were screened as described above. RT-PCR confirmed that the D198K mutation was expressed in the rescued strains without any other sequence modification. Forward swimming velocity was recorded and measured as previously described [6]. For transformations with the control DRC4-GFP plasmid, rescued colonies were recovered at a frequency of 5–15%

### Generation of the Gas8^AV^ mouse allele

CRISPR/sgRNA target sequences were queried using the MIT CRISPR server. Three sites most proximal to the desired SNP change were selected to test nuclease efficiency. CRISPR1: 5’- CTTCTCCACAGCAGCGTTCA *GGG*-3’ (reverse strand), CRISPR2: 5’-GGTGCTGGCCGCCTCCAACC *TGG*-3’ (forward strand), CRISPR3: 5’-GACACAAGCGTTAATGCTGT*GGG*-3’ (reverse strand). Pronuclear injections were performed with Cas9 mRNA (100 ng/ul), CRISPR3/sgRNA (50 ng/ul) and ssODN (200 ng/ul). Efficiency of nuclease activity was assessed using a blastocyst assay. In brief, injected zygotes were cultured to the blastocyst stage and lysed to obtain genomic DNA. Genomic DNA was used in PCR and the amplicons (215 bp) were resolved by heteroduplex mobility assay (HMA). CRISPR3 was found to be most efficient and was used to generate the SNP edited mouse (C57Bl/6 background). Injected zygotes were cultured to 2-cell stage in KSOM mixed with the NHEJ inhibitor SCR7 at a final concentration of 10 mM. The 2-cell stage embryos were transferred to psuedopregnant recipient female mice, which gave birth to 13 pups. The SNP was introduced with the help of a 154 nt single stranded oligo DNA (ssODN) HDR template. Since the PAM sequence (CCC>Pro) could not be modified without changing the amino acid, multiple silent changes were made in the protospacer (sgRNA binding) sequence (indicated by small letters in the sequence below). These changes were made to eliminate the chances of the sgRNA binding to the repaired allele. The SNP change introduced a restriction enzyme recognition enzyme site (BsmBI/Esp3I) and the silent changes introduced two new restriction enzyme recognition sites (BtgI and HaeII). HaeII sites were used to distinguish the wildtype and the modified alleles. Specific primers were also designed that can preferentially amplify the modified allele. HDR template (ssODN) 5’-GGCCCTGAACGCTGCTGTGGAGAAGAGAGAGGTTCAGTTCAATGAGGTGCTGGCCG**T**CTCCAACCTGGACCCCACgGCgcTgACGtTgGTGTCCCGCAAACTTGAGGTAGGTGCCCTCCTGTCCTGTGCTGTGGTACGCCTTCTTGGGTGGCAC-3’. After the initial characterization of the F0 litter by PCR, the 215 bp amplicons were cloned, and selected individual clones were subjected to Sanger sequencing. Sequence analysis of the 13 pups revealed that 2 had complete knock-in of the edited/repair sequence, 1 pup had incorporated the silent changes but did not have the desired SNP change, and 1 pup had indels. F0 animals were bred with wildtype C57Bl/6 mice to test germline transmission of the desired alleles. All alleles were successfully transmitted through the germline, and the positive F1 animals were used to create homozygous and compound heterozygous F2 animals.

### Statistical analysis

Cilia length analysis, bead flow tracking, cilia orientation, and cilia beat frequency were tested with Student’s t-test and graphed in Microsoft Excel. Smoothened trafficking assay and Chlamydomonas swim speeds were tested by ANOVA followed by Student’s t-test with a Bonferroni correction and graphed in Microsoft Excel. All error is represented in Standard Error of Means (SEM).

## Supporting Information

S1 Fig DRC4-DK-GFP is expressed and localizes to the axoneme. Western Blot of *Chlamydomonas* flagellar axonemes showing that *pf2* cells transformed with DRC4-DK-GFP (DK) have proper localization of the protein product. The DRC4-GFP fusion proteins are detected by a DRC4 antibody (top panel) and a GFP antibody (bottom panel). An IC69 antibody against an outer arm dynein intermediate chain serves as a loading control for the blot (bottom panel).
(TIF)

S1 Movie Cilia in Gas8^WT^ trachea display normal beat frequency and waveform (Captured at 240 fps and played back at 30 fps). (AVI)

S2 Movie Cilia in Gas8^GT^ trachea display slower beat frequency and a rigid waveform (Captured at 240 fps and played back at 30 fps). (AVI)
