# Supplementary figures and images for: Mutation of Growth Arrest Specific 8 Reveals a Role in Motile Cilia Function and Human Disease
Source: PLoS Genet. 2016 Jul 29;12(7):e1006220. doi: 10.1371/journal.pgen.1006220 (PMC4966937; doi:10.1371/journal.pgen.1006220)

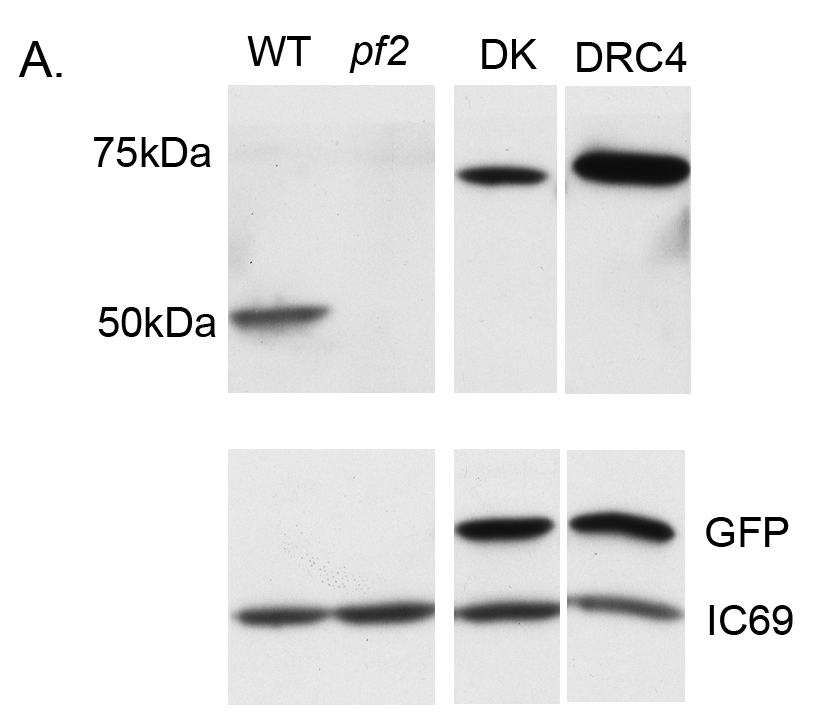

Supplement: S1 Fig — Western Blot of Chlamydomonas flagellar axonemes showing that pf2 cells transformed with DRC4-DK-GFP (DK) have proper localization of the protein product. The DRC4-GFP fusion proteins are detected by a DRC4 antibody (top panel) and a GFP antibody (bottom panel). An IC69 antibody against an outer arm dynein intermediate chain serves as a loading control for the blot (bottom panel). (TIF) [file pgen.1006220.s001.tif]
